# The Royal Orthopædic Hospital

**Published:** 1902-08-16

**Authors:** 


### THE YEAR'S WORK.

### THE ROYAL ORTHOPÆDIC HOSPITAL:

### THE YEAR'S WORK.

Two hundred and fifty-five new in-patients were admitted during 1901, making, with 45 remaining in the wards from the previous year, a total of 300. Of these, 260 were discharged, cured or improved, four were unrelieved or transferred, and 36 remained on December 31st. The daily average number of beds occupied during the year was 39; two wards have, however, been closed since midsummer to reduce expenses. This leaves only 40 beds available instead of 50. The average duration of treatment was 48 days, and the number admitted was 53 more than in 1900. In the out-patients' department there has also been an increase; the number of new patients was 1,060, and of attendances 4,390, an increase of 159 and 462 respectively. The total number of patients treated since the foundation of the hospital in 1838 is 84,458. The average duration of treatment in the wards has shown a steady decrease during the last five years; the following are the figures given in the surgical report: 1897, 101 days; 1898, 93 days; 1899, 70 days; 1900, 67 days; last year, as stated above, 48 days.

In revenue, there was a total loss of nearly £100 on subscriptions and donations, in comparison with the amounts received in the previous year. Subscriptions in 1901 were £438; donations, £475. King Edward's Fund gave £200, the Sunday Fund £95, and the Saturday Fund £44 11s. Invested property realised £377; patients' payments £182 15s. 6d., and Poor-law payments £59 14s. 6d. Other small sums brought the total ordinary income up to £1,879 9s. Legacies, and a special donation for new cots throughout the infants' ward, amounted to £148. The ordinary expenditure was £2,818 15s. 2d.; and under Extraordinary Expenditure appears "Loss of money by burglary, £30."

There has been a change of matron; Miss Mary Pinsent, formerly senior sister, has succeeded Miss Wamsley, who resigned last summer.

